# Herbal Formula Yi‐Fei‐Jie‐Du‐Tang Regulates Epithelial‐Mesenchymal Transition and Vasculogenic Mimicry in Lung Cancer via HIF1A‐Mediated Ferroptosis

**DOI:** 10.1002/adbi.202400306

**Published:** 2025-02-06

**Authors:** Shanshan Wang, Die Yang, Chengjia Yuan, Yang Wu, Qingying Wang, Yongjian Wu, Xiaochun Zhang

**Affiliations:** ^1^ Department of Oncology Yangzhou Hospital of Traditional Chinese Medicine Yangzhou Jiangsu 225002 China; ^2^ Clinical Traditional Chinese Medical College Yangzhou University Yangzhou Jiangsu 225002 China

**Keywords:** cancer, EMT, ferroptosis, hypoxia‐inducible factor, TCM, vasculogenic mimicry

## Abstract

Traditional Chinese medicine (TCM) Yi‐Fei‐Jie‐Du‐Tang (YFJDT) has shown potential in lung cancer treatment. However, the mechanisms underlying the effects of YFJDT on lung cancer remain unclear. Bioinformatics analysis is conducted to identify potential targets of YFJDT. The impact of YFJDT on hypoxia‐inducible factor 1 alpha (HIF1A), ferroptosis, and vasculogenic mimicry (VM) is investigated using xenograft tumor models and A549 cells. Additionally, A549 cells are stimulated with CoCl_2_ to mimic the hypoxic microenvironment of the tumor. The role of HIF1A overexpression in modulating ferroptosis is assessed. The effects of HIF1A and ferroptosis on epithelial‐mesenchymal transition (EMT) and VM in vitro are evaluated. Results: YFJDT treatment led to a concentration‐dependent decrease in HIF1A levels in xenograft tumors and A549 cells. Overexpression of HIF1A counteractes the inhibitory effects of YFJDT on proliferation, EMT, and VM in transplanted tumors. Moreover, HIF1A overexpression attenuates YFJDT‐induced lipid peroxidation and iron accumulation, indicating inhibition of ferroptosis in A549 cells. Hypoxia‐induced alterations in EMT markers and VM are reversed by YFJDT but exacerbated by HIF1A overexpression. Molecular docking identified salicylic acid and psoralen as potential components of YFJDT targeting HIF1A. YFJDT exerts anti‐tumor effects in lung cancer by downregulating HIF1A and promoting ferroptosis.

## Graphical abstract

Yi‐Fei‐Jie‐Du‐Tang (YFJDT), a traditional Chinese medicine, has shown potential in lung cancer treatment by targeting key pathways. This study explores the mechanisms of YFJDT using bioinformatics, xenograft models, and A549 cells, demonstrating that YFJDT downregulates HIF1A, promotes ferroptosis, and inhibits epithelial‐mesenchymal transition (EMT) and vasculogenic mimicry (VM), suggesting novel therapeutic strategies for lung cancer.

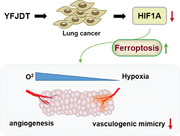

## 1 Introduction

Lung cancer remains one of the most common and lethal malignancies worldwide, posing significant challenges to clinicians and researchers. Lung cancer is an urgent public health problem due to its high morbidity and mortality.^[^ ^1^ ^]^ Current treatment modalities, including surgery, chemotherapy, radiotherapy, and targeted therapy, have improved prognosis to a certain extent.^[^ ^2^ ^]^ However, the prognosis of advanced lung cancer remains poor, mainly due to acquired treatment resistance and metastasis.^[^ ^3^, ^4^ ^]^ Despite advancements in understanding its pathogenesis,^[^ ^5^, ^6^ ^]^ there exists a critical need for novel therapeutic strategies that can effectively target the complex mechanisms underlying lung cancer progression.

Yi‐Fei‐Jie‐Du‐Tang (YFJDT), a traditional Chinese herbal formula, has received increasing attention for its potential anti‐cancer properties, particularly in lung cancer. A previous study by our team has demonstrated its efficacy in inhibiting epithelial‐mesenchymal transition (EMT), a pivotal process implicated in cancer metastasis and therapy resistance.^[^ ^7^ ^]^ Cancer cells undergoing EMT often exhibit increased susceptibility to ferroptosis, a unique form of regulated cell death characterized by iron‐dependent lipid peroxidation.^[^ ^8^, ^9^, ^10^ ^]^ Additionally, emerging evidence suggests that ferroptosis induction has therapeutic potential in overcoming resistance to conventional anti‐cancer therapies.^[^ ^11^, ^12^, ^13^ ^]^ Given these observations, the hypothesis emerged that YFJDT might modulate EMT and ferroptosis pathways, impeding lung cancer progression.

Bioinformatics analysis offers a powerful tool for elucidating the molecular mechanisms underlying the therapeutic effects of Traditional Chinese Medicine.^[^ ^14^ ^]^ Identifying potential targets of YFJDT and its intersection with ferroptosis‐related factors will provide valuable insights into its pharmacological effects. Notably, hypoxia‐inducible factor 1 alpha (HIF1A) is a master regulator of cellular responses to hypoxia, making it a key candidate in this context. A prior study demonstrated that HIF1A promotes lung cancer progression by enhancing invasion and vasculogenic mimicry (VM).^[^ ^15^ ^]^ Additionally, HIF1A has been implicated in the modulation of ferroptosis in liver cancer, further emphasizing its importance in cancer biology.^[^ ^16^ ^]^ Therefore, we speculate that YFJDT may exert its therapeutic effects in lung cancer by modulating HIF1A‐mediated pathways, thus promoting ferroptosis and suppressing EMT and VM.

In summary, this study aims to elucidate the intricate interplay between YFJDT, HIF1A, and ferroptosis in the context of lung cancer progression. Utilizing tumor‐bearing mouse models and A549 cells, we strive to validate our hypothesis and pave the way for the development of innovative anti‐cancer therapies targeting these interconnected pathways.

## 2 Results

### 2.1 Bioinformatics Analysis Results and the Effects of YFJDT on HIF1A and Ferroptosis in Transplanted Tumors

**Figure 1:**
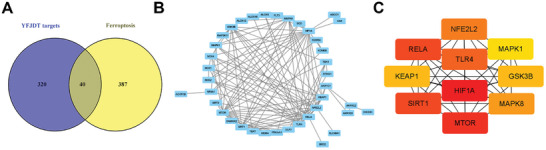
Bioinformatics analysis regarding potential targets of YFJDT. A,B) Bioinformatics analysis results show the intersection between potential targets of YFJDT and factors related to ferroptosis. C) Hub gene analysis reveals the Top 10 genes.

A total of 320 genes were obtained as potential targets of YFJDT, 387 comprehensive factors related to ferroptosis, and 40 intersection genes were obtained (**Figure** **1**A,B). HUB gene analysis resulted in the Top 10 genes, of which the Top 1 with degree value was HIF1A (Figure 1C).

**Figure 2:**
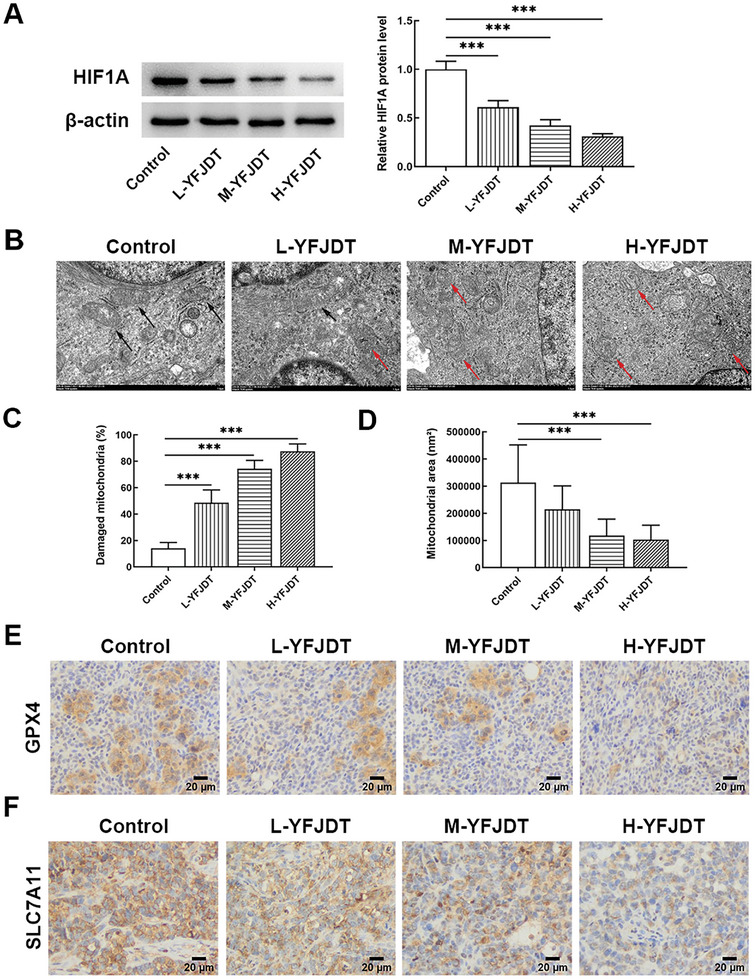
The effects of YFJDT on HIF1A and ferroptosis in transplanted tumors A) Measurement of HIF1A levels in xenograft tumors following treatment with different concentrations of YFJDT. B) Transmission electron microscopy images depicting mitochondrial morphology changes. The black arrows point to representative healthy mitochondria, and the red arrows point to representative damaged mitochondria. According to the results of (B), C) the proportion of damaged mitochondria, and D) the area of mitochondria were analyzed. E) Immunohistochemistry results illustrate the GPX4 and F) SLC7A11 levels following YFJDT treatment. Data are presented as the mean ± S.D., *n* = 3 independent experiments. ***p < 0.001.

HIF1A levels were measured in xenograft tumors of mice given different concentrations of YFJDT. HIF1A levels exhibited a concentration‐dependent decrease, indicating that YFJDT could reduce HIF1A in tumors (**Figure** **2**A). Transmission electron microscopy results revealed that the mitochondria in the control group had normal morphology. Mitochondria are structurally intact and have clear cristae. After the treatment of YFJDT, the mitochondrial cristae were obviously broken or dissolved, and some mitochondria were vacuolated. A small number of mitochondrial membranes even protruded and ruptured in the H‐YFJDT group (Figure 2B,C). The mitochondrial area was generally reduced upon YFJDT treatment (Figure 2D). Immunohistochemistry results displayed that the levels of glutathione peroxidase 4 (GPX4) and solute carrier family 7, membrane 11 (SLC7A11), which block ferroptosis, also showed a concentration‐dependent decrease, indicating that YFJDT has the potential to promote ferroptosis (Figure 2E,F).

### 2.2 Effects of YFJDT, HIF1A, and Ferroptosis on the Malignant Progression of Transplanted Tumors

**Figure 3:**
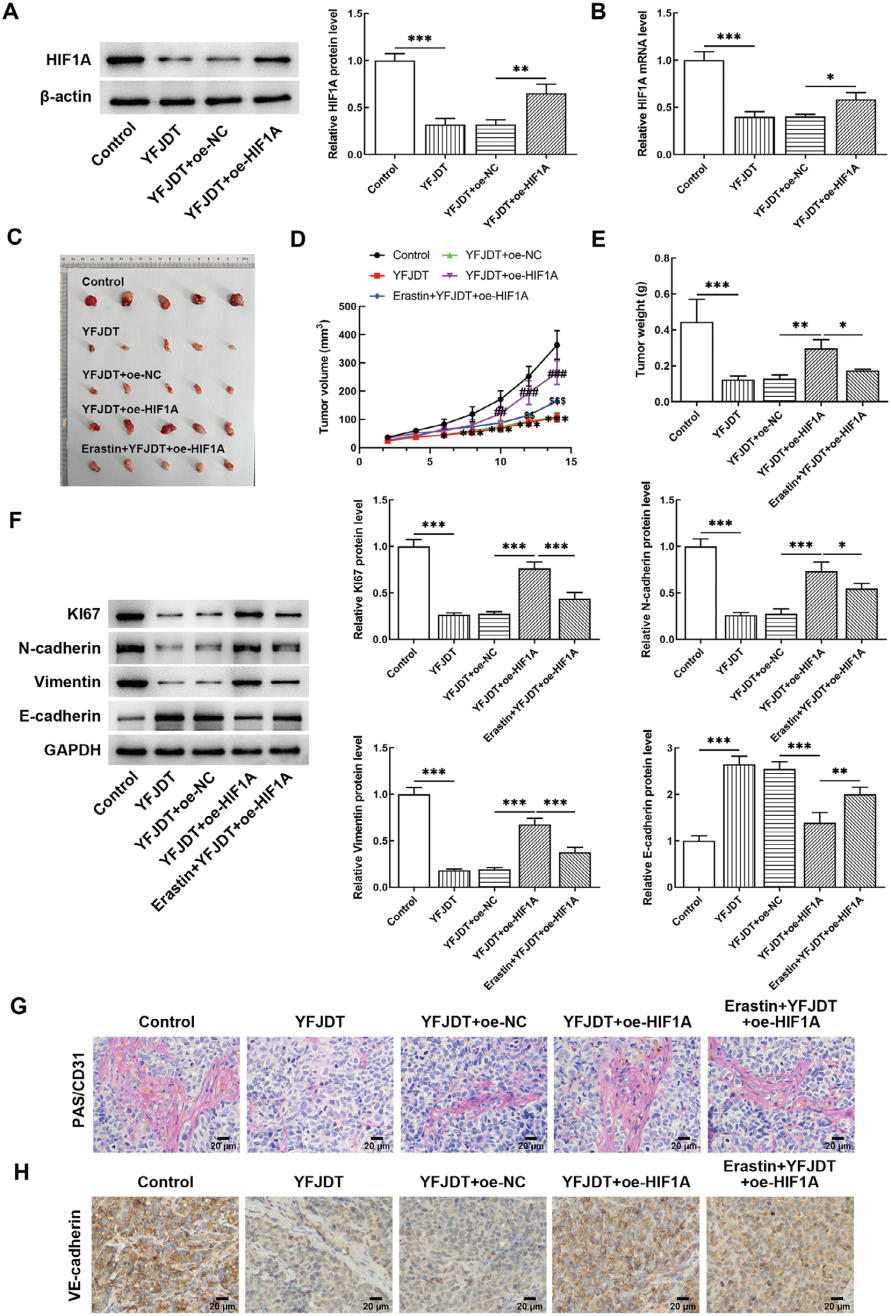
Effects of YFJDT, HIF1A, and ferroptosis on the malignant progression of transplanted tumors. A) HIF1A protein and B) mRNA levels in transplanted tumors formed by normal A549 and transfected cells. C) Photo of the transplanted tumor. D) Tumor volume within 2 weeks. E) Tumor weight. F) Western blot analysis demonstrates the proliferation and epithelial‐mesenchymal transition (EMT) markers. G) PAS/CD31 staining results indicate the endothelium‐dependent and ‐independent angiogenesis. H) Immunohistochemistry results illustrate the VE‐cadherin to indicate vasculogenic mimicry. *p < 0.05, **p < 0.01, ***p < 0.001.

By detecting HIF1A in transplanted tumors formed by injecting cells with overexpression (oe), it was found that compared with the negative control (NC) group, HIF1A remained high in the transplanted tumors (**Figure** **3**A,B). According to the recorded transplanted tumor volume, YFJDT treatment significantly inhibited tumor growth. Compared with YFJDT + oe‐NC, HIF1A overexpression was beneficial to tumor growth, and erastin inhibited tumor growth (Figure 3C,D). These findings were also reflected in tumor weight. YFJDT treatment significantly reduced tumor weight, HIF1A overexpression promoted the increase in weight, and then additional erastin down‐regulated tumor weight (Figure 3E). Western blotting detected the expression levels of proliferation and EMT‐related proteins. YFJDT significantly reduced the levels of Ki67, N‐cadherin, and Vimentin, and increased the level of E‐cadherin, indicating that the proliferation and EMT processes were inhibited. Under the same YFJDT treatment, HIF1A overexpression reversed the change trend of the protein, indicating that reduction of HIF1A is necessary for YFJDT to inhibit proliferation and EMT. Furthermore, additional Erastin inhibited proliferation and EMT, indicating that ferroptosis mediates the regulation of both phenotypes (Figure 3F). HIF1A overexpression broke the reduction of periodic acid schiff (PAS)^+^CD31^−^ region and vascular endothelial (VE)‐cadherin positivity by YFJDT and promoted VM generation. Erastin could reduce VM generation to a certain extent (Figure 3G,H).

### 2.3 Effects of HIF1A Overexpression on Ferroptosis in A549 Cells

**Figure 4:**
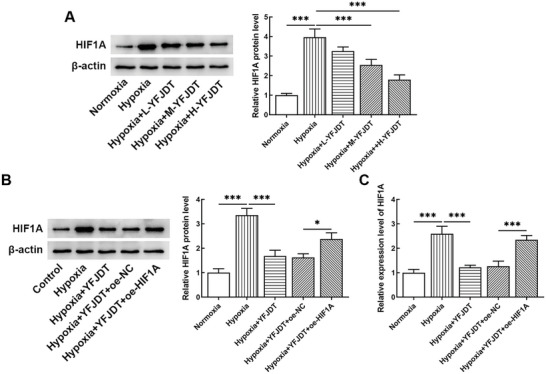
The level of HIF1A in A549 cells A) Evaluation of HIF1A levels in A549 cells under hypoxic conditions and treatment with varying concentrations of YFJDT. B) Analysis of HIF1A protein and C) mRNA levels in A549 cells overexpressing HIF1A and subjected to hypoxia and YFJDT treatment. *p < 0.05, ***p < 0.001.

A549 cells were subjected to hypoxia induction and the effect of YFJDT on HIF1A levels in cells was evaluated. Hypoxia induced an increase in HIF1A levels, and YFJDT inhibited HIF1A levels in a concentration‐dependent manner (**Figure** **4**A). High dose of YFJDT (H‐YFJDT) was used in subsequent experiments. After HIF1A overexpressing cells were treated with hypoxia and YFJDT, HIF1A remained at high levels (Figure 4B,C), which was used to evaluate the impact of HIF1A on ferroptosis in the environment of hypoxia combined with YFJDT.

**Figure 5:**
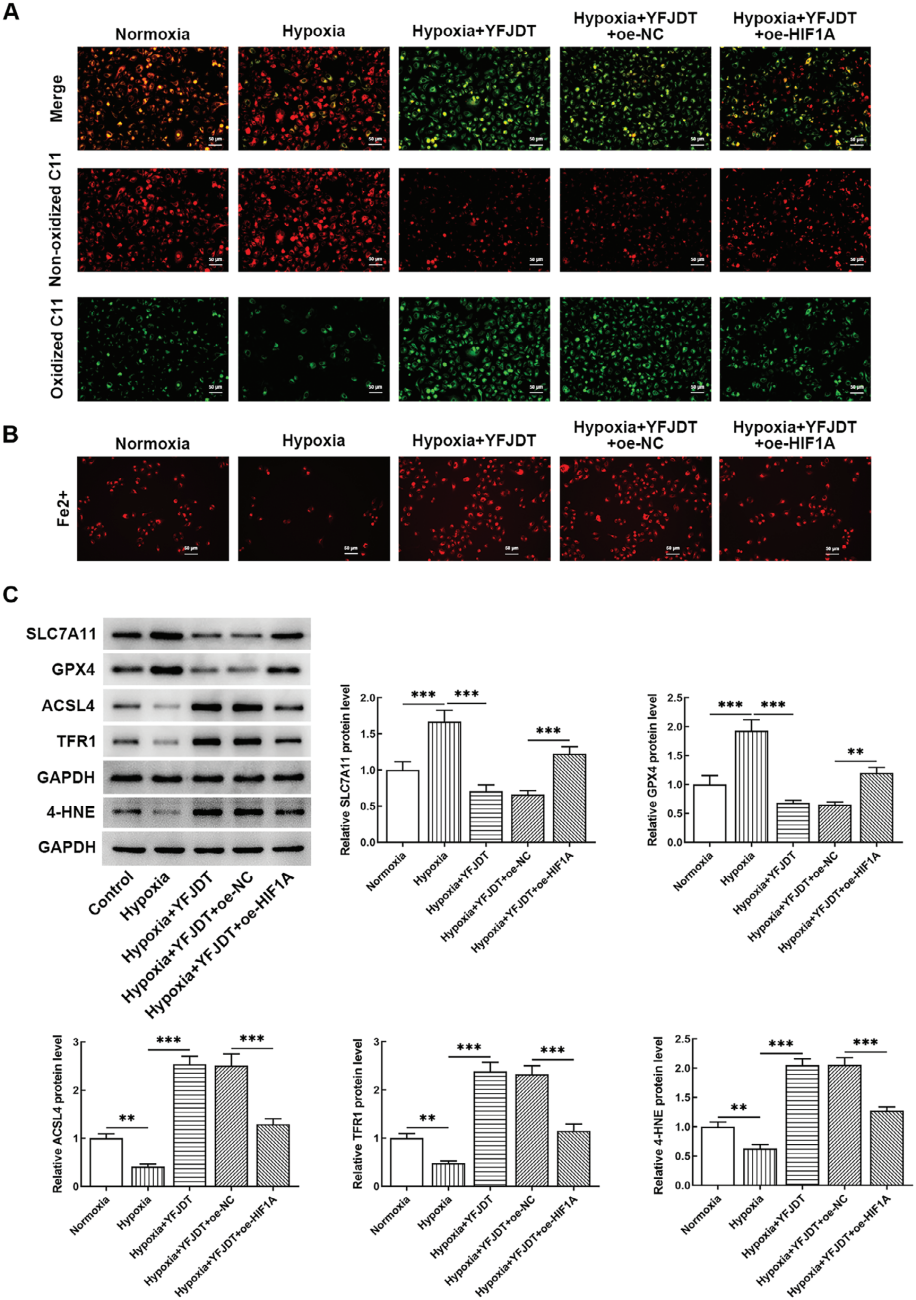
Effects of HIF1A overexpression on ferroptosis in A549 cells A) C11 BODIPY 581/591 results indicate the effects of YFJDT and HIF1A overexpression on lipid peroxidation in A549 cells under hypoxic conditions. B) Measurement of iron levels in A549 cells following treatment with YFJDT and HIF1A overexpression under hypoxic conditions. C) Western blotting analysis shows the impact of YFJDT and HIF1A overexpression on the ferroptosis‐related protein levels in A549 cells under hypoxic conditions. **p < 0.01, ***p < 0.001.

The results of C11 BODIPY 581/591 showed that YFJDT could promote lipid peroxidation, while HIF1A overexpression had an inhibitory effect (**Figure** **5**A). Similarly, HIF1A overexpression reduced YFJDT‐induced increases in iron levels (Figure 5B), as well as acyl‐CoA synthetase long‐chain family 4 (ACSL4), transferrin receptor protein (TFR1), and 4‐hydroxynonenal (4‐HNE) protein levels. In contrast, HIF1A overexpression enhanced the YFJDT‐induced decrease in SLC7A11 and GPX4 protein levels (Figure 5C). These results indicated that upon hypoxic microenvironment, HIF1A overexpression inhibited ferroptosis and reversed the effect of YFJDT.

### 2.4 Effects of YFJDT, HIF1A, and Ferroptosis on Malignant Progression in A549 Cells

**Figure 6:**
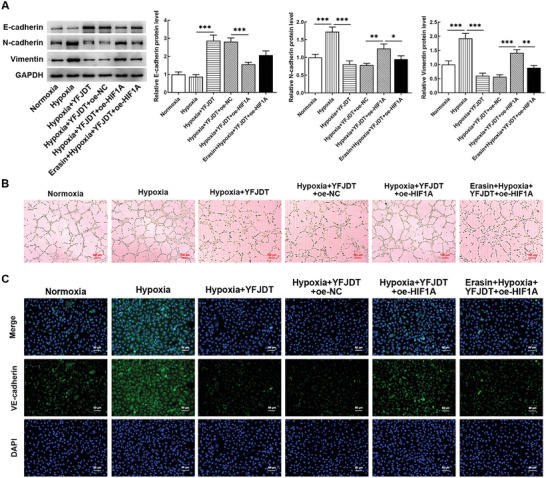
Effects of YFJDT, HIF1A, and ferroptosis on malignant progression in A549 cells A) Western blotting analysis reveals the effects of YFJDT, HIF1A overexpression, and Erastin treatment on the protein levels of EMT markers in A549 cells under hypoxic conditions. B) The effects of YFJDT, HIF1A overexpression, and Erastin treatment on angiogenesis in A549 cells under hypoxic conditions. C) Immunofluorescence results illustrate the impact of YFJDT, HIF1A overexpression, and Erastin treatment on VE‐cadherin levels in A549 cells under hypoxic conditions. *p < 0.05, **p < 0.01, ***p < 0.001.

Hypoxia significantly increased the protein levels of N‐cadherin and Vimentin in cells, but there was no significant difference for E‐cadherin; while YFJDT significantly reversed the protein change trend. HIF1A overexpression significantly decreased E‐cadherin, and increased N‐cadherin and Vimentin protein levels. Erasin significantly reduced N‐cadherin and Vimentin protein levels, accompanied by a slight upregulation of E‐cadherin (**Figure** **6**A). YFJDT significantly reduced hypoxia‐induced angiogenesis, HIF1A overexpression promoted angiogenesis, and additional Erasin treatment inhibited angiogenesis (Figure 6B). Immunofluorescence results showed that YFJDT significantly reduced the hypoxia‐induced increase in VE‐cadherin levels, HIF1A overexpression significantly increased fluorescence, and the addition of Erasin weakened the fluorescence (Figure 6C).

### 2.5 Potential Components in YFJDT that Mainly Act on HIF1A

**Figure 7:**
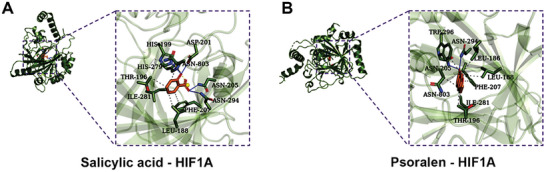
Potential components in YFJDT that mainly act on HIF1A Molecular docking results indicate the binding of salicylic acid and psoralen, components of YFJDT, to HIF1A. Solid blue lines represent hydrogen bonds.

The main compound of YFJDT was molecularly docked with HIF1A. The results revealed that salicylic acid and psoralen in YFJDT could bind to HIF1A, suggesting that these two components might play a major role in the studied phenotypes. In the docking results, the oxygen atom attached to the six‐membered ring in salicylic acid forms hydrogen bonds with the hydrogen atoms of ─N─H in the amino acids HIS and ASN, and also forms a hydrogen bond with the hydrogen atom of −OH in ASP (**Figure** **7**A). The oxygen atom in psoralen forms hydrogen bonds with the hydrogen atoms of ─N─H in the two amino acids ASN (Figure 7B).

## 3 Discussion

Lung cancer is a multifaceted disease characterized by complex interactions between tumor cells and the tumor microenvironment, particularly in hypoxic regions.^[^ ^18^ ^]^ The hypoxic microenvironment within tumors not only promotes tumor‐invasive behavior but also leads to treatment resistance and poor prognosis.^[^ ^19^, ^20^ ^]^ Our study elucidates the therapeutic potential of YFJDT in alleviating EMT and VM in lung cancer. Both in vivo experiments using tumor‐bearing mouse models and in vitro studies with A549 cells demonstrated the efficacy of YFJDT in inhibiting these crucial pathways.

Furthermore, our study reveals the critical role of HIF1A in mediating EMT and VM in lung cancer. We observed that HIF1A overexpression promoted EMT and VM, partially reversing the therapeutic effects of YFJDT. Of note, similar observations regarding the role of HIF1A in driving tumor progression have been reported in other cancers. For instance, in breast cancer, HIF1A facilitates angiogenesis by inducing tumor and stromal cells to secrete pro‐angiogenic factors.^[^ ^19^ ^]^ Activation of HIF1A transcription under hypoxic conditions promotes stemness and chemoresistance in colorectal cancer.^[^ ^21^ ^]^ In contrast, conflicting evidence suggests that pancreatic‐specific HIF1A deletion accelerates the progression of pancreatic ductal adenocarcinoma.^[^ ^22^ ^]^ These conflicting findings highlight the complexity of HIF1A signaling in cancer, likely involving feedback mechanisms and intertumor heterogeneity,^[^ ^23^ ^]^ necessitating further investigations.

Moreover, our study delineated the interplay between HIF1A and ferroptosis upon YFJDT treatment in regulating lung cancer progression. The inhibition of EMT and VM was found in both transplanted tumors and A549 cells was associated with induction of ferroptosis. Ferroptosis, an iron‐dependent form of cell death, has emerged as a promising therapeutic avenue in cancer treatment.^[^ ^24^ ^]^ Unlike apoptosis or necrosis, ferroptosis is characterized by the accumulation of lipid peroxides and can be induced by various stimuli, including oxidative stress and metabolic dysregulation.^[^ ^25^ ^]^ Importantly, ferroptosis induction holds therapeutic potential in overcoming resistance to EMT‐driven cancers.^[^ ^8^, ^26^, ^27^ ^]^ Previous studies have implicated HIF1A in modulating ferroptosis sensitivity, wherein HIF1A‐induced lactate production promotes ferroptosis resistance in a pH‐dependent manner. Silencing HIF1A sensitizes solid tumors to ferroptosis induction.^[^ ^28^ ^]^ These findings suggest that targeting HIF1A‐mediated pathways may be a promising strategy to enhance ferroptosis in lung cancer. However, YFJDT exerts its therapeutic effects through multiple bioactive compounds, possibly targeting different pathways and biological processes. The inhibitory effect of YFJDT on HIF1A is considered to be that some active ingredients suppress the transcription of the HIF‐1α gene or affect the stability of its mRNA. Additionally, HIF1A is a protein that is easily degraded. Some ingredients may increase the hydroxylation of HIF1A and promote its binding to the von Hippel‐Lindau protein, thereby accelerating the degradation of HIF1A through the ubiquitin‐proteasome pathway.^[^ ^29^ ^]^ Although this study ultimately focused on HIF1A and proposed two potential primary compounds, identifying the specific compounds responsible for the observed effects was challenging and a limitation of this study.

In conclusion, this study elucidates the therapeutic potential of YFJDT to modulate EMT and angiogenic mimicry in lung cancer by promoting ferroptosis through HIF1A. These findings contribute to a deeper understanding of the molecular mechanisms of lung cancer progression and provide insights for the development of new therapeutic strategies targeting these interconnected pathways. Further investigation into the precise mechanisms controlling HIF1A‐mediated ferroptosis may reveal additional therapeutic targets to combat lung cancer and overcome treatment resistance.

## 4 Experimental Section

### Bioinformatics Analysis

Following the findings from our previously published study utilizing high‐performance liquid chromatography (HPLC),^[^ ^7^ ^]^ seven chemical constituents including chlorogenic acid, salicylic acid, hyperoside, etc. were selected for network pharmacological analysis. Employing SwissTargetPrediction, TargetNet, and Super‐PRED, accessible through the respective URLs (swisstargetprediction.ch, targetnet.scbdd.com, prediction.charite.de), a total of 320 genes were obtained. Additionally, a comprehensive set of 387 ferroptosis‐related factors was retrieved from the FerrDB database (zhounan.org/ferrdb). Intersectional analysis between the compound‐targeted genes and ferroptosis‐associated factors was conducted. Subsequently, HUB gene analysis was undertaken utilizing the String database in conjunction with Cytoscape 3.9.0 software, facilitating the identification of the Top10 genes. The compounds targeting HIF1A underwent molecular docking analysis utilizing AutoDock 4 software. Briefly, the structure of HIF1A was downloaded from the Protein Data Bank (rcsb.org) and prepared by removing excess water molecules and adding hydrogen atoms. The 3D structure of chemical compounds was downloaded from PubChem (pubchem.ncbi.nlm.nih.gov) and hydrogenated. The molecular docking module in the software was run and the results were exported.

### Cell Culture and Transfection

Human A549 cells (Cell Bank, Chinese Academy of Sciences, Shanghai) were cultured in the RPMI‐1640 medium in an incubator with 5% CO_2_ at 37 °C. X‐tremeGENE transfection reagent (MilliporeSigma) and pcDNA plasmids carrying HIF1A (Honorgene, Changsha) were added to the cells, and then they were incubated for 48 h. Those transfected with empty plasmid were used as the negative control.

### Establishment of the Tumor‐Bearing Mouse Model

BALB/c male nude mice (20–24 g, Center for Disease Control and Prevention, Jiangsu) were raised normally to adapt to the environment and had free access to feed and purified water. The preparation of YFJDT refers to the previous article published by our team.^[^ ^7^ ^]^ A549 cells and transfected cells were made into a suspension, and the suspension (0.1 mL, 1×10^7^ mL^−1^) was injected into the flank of nude mice. Initially, tumor‐bearing nude mice were randomly divided into four groups: control group (normal saline, i.g.), YFJDT low, middle, and high‐dose groups (YFJDT 9, 18, 36 g/kg/day, i.g.). After evaluating the basic drug efficacy, the concentration of YFJDT was selected. The nude mice were then divided into five groups, with six mice in each group, and transfected cells (oe‐NC and oe‐HIF1A) were injected to form transplanted tumors, along with injection of the ferroptosis inducer Erastin (10 µL g^−1^ day^−1^, i.p.).^[^ ^17^ ^]^ The volume of the transplanted tumors was measured every two days, and the mice were euthanized after two weeks and the transplanted tumors were isolated and weighed.

### Cell Treatment

A549 cells (1 × 10^6^ cells mL^−1^) in serum‐free RPMI‐1640 medium were supplemented with 150 µmol L^−1^ of CoCl_2_ to mimic the hypoxic microenvironment of the tumor. Blood was collected from the abdominal aorta 1 h after the last administration of YFJDT to the mice, and the serum was separated. The serum was filtered through a 0.22 micropore filter, sterilized, and stored in a −80 °C refrigerator. Cells were incubated with YFJDT‐containing serum in a hypoxic environment for 24 h.

### Western Blot

The total proteins were extracted from A549 cells or tissues using 1 × RIPA lysis buffer and the concentrations of extracted proteins were measured by the BCA method. Equal amounts of proteins were then separated by SDS‐PAGE and transferred to membranes. After blocking with 5% non‐fat milk for 1 h, the membranes were incubated at 4 °C overnight with the primary antibodies against HIF1A (A22041), Ki67 (A16919), E‐cadherin (A22850), N‐cadherin (A3045), vimentin (A19607), SLC7A11 (A25302), GPX4 (A1933), ACSL4 (A20414), TFR1 (TFRC, A5865), GAPDH (A19056), β‐actin (AC048, all from ABclonal), and 4HNE (5215, KeyGen Biotech). Subsequently, the membranes were probed with secondary antibodies (AS014, ABclonal) at room temperature for 1 h. Protein bands were visualized and quantified using ImageJ software.

### Transmission Electron Microscope

Fresh tissue was immersed with 2.5% glutaraldehyde and postfixed with 1% OsO4 for 2 h. After dehydration with gradient ethanol and embedding in resin with acetone, 50 nm‐thick sections were cut from the polymerized blocks. The sections were stained with uranium acetate for 20 min and lead citrate for 10 min before visualization of mitochondrial morphology using transmission electron microscope (Hitachi) at 80 kV.

### Immunohistochemistry

Tumor tissue sections embedded in paraffin were sliced into 4 µm‐thick sections after fixation with 4% formaldehyde. Deparaffinized sections underwent antigen retrieval with 10 mm citrate buffer. Endogenous peroxidase was blocked with 3% hydrogen peroxide for 10 min before overnight incubation at 4 °C with primary antibodies against GPX4 (ABclonal), SLC7A11 (ABclonal), and VE‐cadherin (ab318152, Abcam). Subsequent incubation with HRP goat anti‐rabbit IgG (PV‐6001, ZSGB‐BIO) for 20 min at room temperature was followed by DAB staining and hematoxylin counterstaining. Images were captured under a microscope (Leica, Germany).

### RT‐qPCR

Total RNA was extracted with TRIzol reagent (Life Technologies) from A549 cells. Afterward, total RNA (500 ng) was reverse‐transcribed using a High‐Capacity cDNA Reverse Transcription Kit (4 368 814, Applied Biosystems). QRT‐PCR assay was performed with cDNA, primers mix, and SYBR Green Master (QIAGEN) by a 7500 Fast Real‐Time instrument (Applied Biosystems, USA). The relative expression level was normalized to the beta‐actin gene.

### PAS / CD31 Staining

Sections were subjected to immunohistochemistry, using CD31antibody (ab76533, Abcam) to visualize blood endothelial cells. Next, sections were incubated with periodic acid solution for 5 min and Schiff reagent for 15 min, followed by staining with hematoxylin. Images were captured under a microscope (Leica, Germany).

### BODIPYC11 and Fe2+ Detection

C11 BODIPY 581/591 probe (RM02821, ABclonal) served as an indicator of lipid peroxidation. FeRhoNox‐1 probe (FS1348, Fushen biotech, Shanghai) was used to reflect intracellular ferrous iron (Fe^2+^). Diluted probe solution (5 µm) was added to the wells, followed by incubation for 30 min. After washing with phosphate buffered saline (PBS)IF, cells were observed under a fluorescence microscope (Leica, Germany).

### Tube Formation Experiment

96‐well plates were coated with matrigel and left at 37 °C for 1 h. A549 cell suspensions (2×10^4^ cells well^−1^) were seeded on the matrigel. Representative images at 8 h‐point were taken by a microscope (Leica, Germany).

### Immunofluorescence

A549 cells underwent washes with PBS, fixation with 4% paraformaldehyde for 20 min, and permeabilization with 0.1% Triton X‐100. Subsequently, the cells were exposed to a VE‐cadherin primary antibody (Abcam) overnight at 4 °C, succeeded by incubation with FITC‐conjugated goat anti‐rabbit antibody (AS011, ABclonal) for 1 h in the absence of light. Following this, the cells were counterstained with DAPI for 5 min. Representative images were obtained under a fluorescence microscope (Leica, Germany).

### Statistical Analysis

Statistical analysis was performed using GraphPad Prism 8.02 and data are presented as mean ±  standard deviation from independent bio‐triplications. One‐way analysis of variance (ANOVA) followed by Tukey's post‐hoc correction was applied for comparison. *P* < 0.05 was considered significant.

## Conflict of Interest

The author declares no conflict of interest.

## Author Contributions

S.W., D.Y., and X.Z. contributed to the conception design, analysis, and interpretation of the data. C.Y., Y.W. Q.W., and Y.W. contributed to the investigation, acquisition, analysis, and interpretation of data. S.W. drafted the article and all authors commented on previous versions of the manuscript. All authors approved the final version of the article.

## Data Availability

The datasets used and/or analyzed during the current study are available from the corresponding author on reasonable request.

## References

[1] M. B. Schabath , M. L. Cote , Cancer Epidemiol Biomarkers Prev. 2019, 28, 1563. 31575553 10.1158/1055-9965.EPI-19-0221 PMC6777859

[2] R. Nooreldeen , H. Bach , Int. J. Mol. Sci. 2021, 22, 8661. 34445366 10.3390/ijms22168661 PMC8395394

[3] J. H. Lee , A. Saxena , G. Giaccone , Semin. Cancer Biol. 2023, 93, 123. 37236329 10.1016/j.semcancer.2023.05.008

[4] M. Wang , R. S. Herbst , C. Boshoff , Nat. Med. 2021, 27, 1345. 34385702 10.1038/s41591-021-01450-2

[5] I. Toumazis , M. Bastani , S. S. Han , S. K. Plevritis , Lung Cancer 2020, 147, 154. 32721652 10.1016/j.lungcan.2020.07.007

[6] A. K. P. Ganti , B. W. Loo , M. Bassetti , C. Blakely , A. Chiang , T. A. D'Amico , C. D'Avella , A. Dowlati , R. J. Downey , M. Edelman , C. Florsheim , K. A. Gold , J. W. Goldman , J. C. Grecula , C. Hann , W. Iams , P. Iyengar , K. Kelly , M. Khalil , M. Koczywas , R. E. Merritt , N. Mohindra , J. Molina , C. Moran , S. Pokharel , S. Puri , A. Qin , C. Rusthoven , J. Sands , R. Santana‐Davila , et al., J Natl Compr Canc Netw. 2021, 19, 1441. 34902832 10.6004/jnccn.2021.0058 PMC10203822

[7] S. Wang , Z. Wang , Y. Wu , et al., Evid Based Complement Alternat Med. 2022, 2022, 9160616. 35132327 10.1155/2022/9160616 PMC8817838

[8] N. Ebrahimi , S. Adelian , S. Shakerian , M. Afshinpour , S. R. Chaleshtori , N. Rostami , F. Rezaei‐Tazangi , S. Beiranvand , M. R. Hamblin , A. R. Aref , Cytokine Growth Factor Rev. 2022, 64, 33. 35219587 10.1016/j.cytogfr.2022.01.006

[9] V. S. Viswanathan , M. J. Ryan , H. D. Dhruv , S. Gill , O. M. Eichhoff , B. Seashore‐Ludlow , S. D. Kaffenberger , J. K. Eaton , K. Shimada , A. J. Aguirre , S. R. Viswanathan , S. Chattopadhyay , P. Tamayo , W. S. Yang , M. G. Rees , S. Chen , Z. V. Boskovic , S. Javaid , C. Huang , X. Wu , Y.‐Y. Tseng , E. M. Roider , D. Gao , J. M. Cleary , B. M. Wolpin , J. P. Mesirov , D. A. Haber , J. A. Engelman , J. S. Boehm , J. D. Kotz , et al., Nature 2017, 547, 453. 28678785 10.1038/nature23007 PMC5667900

[10] M. J. Hangauer , V. S. Viswanathan , M. J. Ryan , D. Bole , J. K. Eaton , A. Matov , J. Galeas , H. D. Dhruv , M. E. Berens , S. L. Schreiber , F. McCormick , M. T. McManus , Nature 2017, 551, 247. 29088702 10.1038/nature24297 PMC5933935

[11] X. Jiang , B. R. Stockwell , M. Conrad , Nat. Rev. Mol. Cell Biol. 2021, 22, 266. 33495651 10.1038/s41580-020-00324-8 PMC8142022

[12] Y. Wang , X. Wu , Z. Ren , Y. Li , W. Zou , J. Chen , H. Wang , Drug Resist. Updat. 2023, 66, 100916. 36610291 10.1016/j.drup.2022.100916

[13] C. Zhang , X. Liu , S. Jin , Y. Chen , R. Guo , Mol Cancer 2022, 21, 47. 35151318 10.1186/s12943-022-01530-y PMC8840702

[14] L.i Zhao , H. Zhang , N. Li , J. Chen , H. Xu , Y. Wang , Q. Liang , J. Ethnopharmacol. 2023, 309, 116306. 36858276 10.1016/j.jep.2023.116306

[15] R. Fu , W. Du , Z. Ding , Y.i Wang , Y. Li , J. Zhu , Y. Zeng , Y. Zheng , Z. Liu , J.‐A. Huang , Cell Death Dis. 2021, 12, 394. 33850110 10.1038/s41419-021-03682-z PMC8044151

[16] Z. Fan , G. Yang , W. Zhang , Q. Liu , G. Liu , P. Liu , L. Xu , J. Wang , Z. Yan , H. Han , R. Liu , M. Shu , J. Cell. Mol. Med. 2021, 25, 10197. 34609072 10.1111/jcmm.16957 PMC8572766

[17] X. F. Jie , Y. P. Li , S. Liu , Y. Fu , Y. Y. Xiong , Biochem. Biophys. Res. Commun. 2023, 671, 309. 37327702 10.1016/j.bbrc.2023.05.057

[18] T. W. Battaglia , I. L. Mimpen , J. J. H. Traets , A. van Hoeck , L. J. Zeverijn , B. S. Geurts , G. F. de Wit , M. Noë , I. Hofland , J. L. Vos , S. Cornelissen , M. Alkemade , A. Broeks , C. L. Zuur , E. Cuppen , L. Wessels , J. van de Haar , E. Voest , Cell 2024, 187, 2324. 38599211 10.1016/j.cell.2024.03.021

[19] E. C. de Heer , M. Jalving , A. L. Harris , J. Clin. Invest. 2020, 130, 5074. 32870818 10.1172/JCI137552 PMC7524491

[20] Y. Zhang , X. Liu , L. Zeng , X. Zhao , Q. Chen , Y. Pan , Y. Bai , C. Shao , J. Zhang , Br. J. Cancer 2022, 127, 1760. 36050447 10.1038/s41416-022-01956-7 PMC9643351

[21] M. Mu , Q. Zhang , J. Li , C. Zhao , X. Li , Z. Chen , X. Sun , J. Yu , Cell Death Differ. 2023, 30, 2393. 37816999 10.1038/s41418-023-01228-8 PMC10657471

[22] A. Tiwari , K. Tashiro , A. Dixit , A. Soni , K. Vogel , B. Hall , I. Shafqat , J. Slaughter , N. Param , A. Le , E. Saunders , U. Paithane , G. Garcia , A. R. Campos , J. Zettervall , M. Carlson , T. K. Starr , Y. Marahrens , A. J. Deshpande , C. Commisso , P. P. Provenzano , A. Bagchi , Gastroenterology 2020, 159, 1882. 32768595 10.1053/j.gastro.2020.07.046 PMC7680408

[23] N. M. Anderson , M. C. Simon , Curr. Biol. 2020, 30, R921. 32810447 10.1016/j.cub.2020.06.081 PMC8194051

[24] N. Xing , Q. Du , S.a Guo , G. Xiang , Y.i Zhang , X. Meng , L.i Xiang , S. Wang , Cell Death Discov 2023, 9, 110. 37005430 10.1038/s41420-023-01407-z PMC10067943

[25] K. Newton , A. Strasser , N. Kayagaki , V. M. Dixit , Cell death 2024, 187, 235. 10.1016/j.cell.2023.11.044 38242081

[26] Y. Ren , X. Mao , H. Xu , Q. Dang , S. Weng , Y. Zhang , S. Chen , S. Liu , Y. Ba , Z. Zhou , X. Han , Z. Liu , G. Zhang , Cell. Mol. Life Sci. 2023, 80, 263. 37598126 10.1007/s00018-023-04907-4 PMC10439860

[27] H. Zhang , N. Chen , C. Ding , H. Zhang , D. Liu , S. Liu , Front. Oncol. 2024, 14, 1344290. 38469234 10.3389/fonc.2024.1344290 PMC10926930

[28] Z. Yang , W. Su , X. Wei , S. Qu , D. Zhao , J. Zhou , Y. Wang , Q. Guan , C. Qin , J. Xiang , K.e Zen , B. Yao , Cell Rep. 2023, 42, 112945. 37542723 10.1016/j.celrep.2023.112945

[29] G. L. Semenza , Curr. Opin. Cell Biol. 2001, 13, 167. 11248550 10.1016/s0955-0674(00)00194-0

